# HPV-independent squamous sell carcinoma of cervix: a clinicopathological, immunohistochemical, and molecular analysis of six cases

**DOI:** 10.1007/s00428-025-04113-6

**Published:** 2025-05-10

**Authors:** Gözde Kır, Ahmet Erbağcı, Filiz Özen, Diyar SAYIT, Ateş Karateke

**Affiliations:** 1 Department of Pathology, Istanbul Medeniyet University, Eğitim Mah. Fahrettin Kerim Gökay Caddesi, Kadıköy, Istanbul, 34722 Turkey; 2 Department of Medical Genetics, Istanbul Medeniyet University, Eğitim Mah. Fahrettin Kerim Gökay Caddesi, Kadıköy, Istanbul, 34722 Turkey; 3 Private Practice, Istanbul, Turkey

**Keywords:** HPV-independent, Cervical squamous cell carcinoma, Immunohistochemistry, Molecular pathology, Next-generation sequencing

## Abstract

HPV-independent squamous cell carcinoma (SCC) of the cervix is a rare subtype associated with aggressive clinical behavior and poor prognosis, as reported in limited studies with small sample sizes. This study aims to investigate the clinicopathological, immunohistochemical, and molecular characteristics of six cases of HPV-independent SCC. Formalin-fixed, paraffin-embedded tissues from six patients were analyzed using immunohistochemistry and Next-Generation Sequencing (NGS). Human papillomavirus (HPV) testing was performed using the Aptima HPV assay, and immunohistochemical markers, including p16, p53, PD-L1, HER2 and CK5/6 were evaluated. The median age at diagnosis was 62 years. Key histopathological features included an infiltrative-destructive growth pattern, pronounced keratinization, and extensive necrosis in the majority of cases. All tumors were p16-negative and high-risk HPV-negative by the Aptima HPV assay, confirming their HPV-independent status. One case exhibited p53 overexpression and HER2 positivity (score 3 +). Five out of six patients succumbed to the disease. FIGO stages ranged from IIB to IVB. Molecular analysis identified mutations in TP53 and PIK3CA, as well as novel CTNNB1 and TERT promoter mutations, and HER2 amplification**.** In five of six patients PD-L1 expression with at least CPS score of 2,5 was observed. HPV-independent cervical SCC typically affects older patients and is associated with poor clinical outcomes. This study highlights TP53 and PIK3CA mutations and novel findings, including CTNNB1, TERT promoter mutations, and HER2 amplification. The observed PD-L1 expression patterns may suggest potential benefits from immunotherapy. Further research is warranted to better understand the clinicopathologic characteristics and molecular drivers of HPV-independent SCC and to explore novel therapeutic strategies.

**Supplementary Information:**

The online version contains supplementary material available at 10.1007/s00428-025-04113-6.

## Introduction

Cervical cancer remains one of the leading causes of cancer-related morbidity and mortality among women worldwide, with squamous cell carcinoma (SCC) being the most prevalent histological subtype. While the vast majority of cervical SCC cases are associated with high-risk human papillomavirus (HPV) infection, a small but clinically significant subset of cases is HPV-independent. The recognition of HPV-independent SCC as a distinct entity by the World Health Organization (WHO) underscores its unique pathogenesis and clinical behavior [1]. Unlike HPV-associated SCC, HPV-independent SCC typically affects older women, often presenting at more advanced stages, which contributes to a worse overall prognosis [2].

The etiology and molecular drivers of HPV-independent SCC remain poorly understood, presenting significant challenges in both diagnosis and treatment. Studies have demonstrated that these tumors frequently lack p16 expression and negative for HPV DNA or RNA, even when assessed with highly sensitive detection methods [3, 4].

This study aims to contribute to the literature by providing a comprehensive analysis of six cases of HPV-independent cervical SCC, focusing on their histopathological features, immunohistochemical profiles, and molecular alterations. Additionally, we explored the expression of immune markers including HER2 and PD-L1, which may provide insights into potential future treatment strategies.

## Materıals and methods

### Histopathology and ımmunohistochemistry

Biopsy specimens were fixed in 10% formaldehyde and processed into paraffin-embedded tissue using standard techniques. Hematoxylin and Eosin (H&E) staining was performed on 4-μm sections for routine histological examination. H&E-stained slides from all patients were independently reviewed by two pathologists (GK, AE).

For immunohistochemical work four-micrometer sections were cut from formalin-fixed paraffin-embedded (FFPE) blocks and stained using the Benchmark Ultra system (Ventana, Roche) following the manufacturer’s protocol.Details regarding the antibodies used (p16, p53, HER2, CK 5/6 and PD-L1), their sources, catalogue numbers, staining instruments, and clones are provided in Table 1.

**Table 1:** List of immunohistochemical markers

	p16	p53	HER2	PD-L1	CK 5/6
Vendor	Roche	Roche	Roche	Roche	Roche
Staining instrument	Roche Benchmark Ultra	Roche Benchmark Ultra	Roche Benchmark Ultra	Roche Benchmark Ultra	Roche Benchmark Ultra
Dilution	Ready to use	Ready to use	Ready to use	Ready to use	Ready to use
Detection	Optiview	Optiview	Optiview	Optiview	Optiview
Clone	E6H4™	Bp53-11	4B5	SP263	D5/16B4

The interpretation of p16 staining was based on established criteria: block-like staining (diffusely cytoplasmic with at least focal nuclear staining) was considered positive, while no staining or patchy staining was deemed negative [5]. p53 expression was evaluated using published criteria for vulvar SCC, with mutant/aberrant patterns defined as uniformly strong nuclear staining in ≥ 80% of tumor cells or complete absence of staining (with internal positive controls in adjacent inflammatory and stromal cells), or cytoplasmic staining (with or without nuclear staining). In contrast, wild-type p53 expression was identified by heterogeneous nuclear staining of variable intensity within tumor cells [6].

HER2 scoring was conducted following ASCO/CAP guidelines [7]. For PD-L1 assessment, tumor cells and mononuclear inflammatory cells (within tumor nests or adjacent stroma) exhibiting partial or complete linear membrane staining of at least 1 + intensity were considered positive [8]. The expression level was assessed using the combined positive score (CPS), which is calculated as the ratio of PD-L1-positive cells (including tumor cells, lymphocytes, and macrophages) to the total number of tumor cells × 100. PD-L1 positivity was defined as a CPS of 1 or higher [9].

Cytokeratin 5/6 was performed to confirm squamous histotype and exclude secondary cervical involvement by other neoplasms.

### Aptima HPV assay and genotyping

The Aptima HPV assay (Hologic, San Diego, CA, USA) was employed for HPV detection. This transcription-mediated amplification (TMA) assay qualitatively detects E6/E7 mRNA from 14 high-risk HPV types (16, 18, 31, 33, 35, 39, 45, 51, 52, 56, 58, 59, 66, and 68) [10]. The Aptima HPV 16 and HPV 18/45 genotype assay (AHPV-GT; Hologic) was used for type-specific detection of HPV 16 and HPV 18/45. Other high-risk types were grouped as"other."This FDA-approved assay identifies active oncogenic infections and is validated for liquid-based cytology specimens.

### Aptima HPV assay methodology

To prevent nucleic acid cross-contamination, new blades were used for each specimen, and sectioning areas were thoroughly cleaned with 3% sodium hypochlorite, followed by distilled water. For each tissue block, 8–60 (depending on size) 4-μm sections were placed in 1.5 mL microcentrifuge tubes, treated with mineral oil, and incubated at 95 °C for 6 min. After centrifugation, the supernatant was discarded, and the process was repeated three times to obtain a tissue pellet. The pellet was treated with Proteinase K (20 mg/mL) and Aptima sample solution, incubated at 56 °C overnight, and analyzed on the Panther system (Hologic) using the Aptima high-risk HPV assay. Results ≥ 0.5 were considered positive. The protocol was modified and standardized to accommodate FFPE tissue lysates for compatibility with the Aptima system. All procedures adhered to the manufacturer's recommendations [11].

### Mutational analysis

DNA was extracted from primary tumor samples for analysis using the"Archer® VariantPlex® Pan Solid Tumor"kit, designed to detect mutations, indels, structural alterations, and microsatellite instability in 185 genes commonly affected in solid tumors (*full gene list in Supplementary Materials). Amplification was performed using Anchored Multiplex PCR (AMP™), followed by sequencing on the Illumina NextSeq platform [12].

Sequencing data underwent alignment to the hg19 (GRCh37) reference genome and variant detection using Archer Analysis software, which employs molecular barcoding to reduce false positives. Variants with an allele fraction (AF) > 5% were prioritized for reporting, though lower AF variants were included if clinically relevant. Tier III variants were evaluated for relevance to the cancer type and population database frequency, while Tier IV variants (likely benign or benign) were excluded from the report [13].

This comprehensive methodology ensured accurate identification of genetic alterations and provided valuable insights into the molecular underpinnings of the tumors.

*ABL1, ACVR1, AKT1, AKT2, AKT3, ALK, APC, AR, ARID1 A, ARID1B, ARID2, ATM, ATR, ATRX, AURKA, B2M, BAP1, BARD1, BCOR, BLM, BMPR1 A, BRAF, BRCA1, BRCA2, BRIP1, CCND1, CCND2, CCND3, CCNE1, CDH1, CDK12, CDK4, CDK6, CDKN2 A, CDKN2B, CHD1, CHEK1, CHEK2, CIC, CSF1R, CTNNB1, DAXX, DDR2, DDX3X, DICER1, EGFR, EIF1 AX, EP300, EPCAM, ERBB2, ERBB3, ERBB4, ERCC1, ERCC2, ESR1, EZH2, FANCA, FANCI, FANCL, FBXW7, FGF19, FGFR1, FGFR2, FGFR3, FGFR4, FH, FLCN, FLT1, FLT3, FLT4, FOXA1, FOXL2, FUBP1, GNA11, GNAQ, GNAS, H3 F3 A, H3 F3B, HIST1H3B, HIST1H3 C, HNF1 A, HRAS, IDH1, IDH2, JAK1, JAK2, JAK3, KDM6 A, KDR, KEAP1, KIT, KLF4, KMT2 C, KMT2D, KRAS, LZTR1, MAP2 K1, MAP2 K2, MAP3 K1, MDM2, MDM4, MED12, MEN1, MET, MLH1, MPL, MRE11 A, MSH2, MSH3, MSH6, MTOR, MUC16, MUTYH, MYC, MYCN, NBN, NF1, NF2, NKX2-1, NOTCH1, NOTCH2,NOTCH3, NOTCH4, NPM1, NRAS, NTRK1, NTRK2, NTRK3, PALB2, PBRM1, PDGFRA, PIK3 CA,PIK3R1, PLCB4, PMS2, POLD1, POLE, PPP2R1 A, PPP2R2 A, PRKD1, PTCH1, PTEN, PTPN11, RAD50, RAD51, RAD51B, RAD51 C, RAD51D, RAD54L, RAF1, RB1, RET, RHOA, RICTOR, RNF43, ROS1, SDHA, SDHB, SDHC, SDHD, SETD2, SF3B1, SMAD2, SMAD4, SMARCA4, SMARCB1, SMO, SRC, SRSF2, STAG2, STK11, SUFU, TERT, TGFBR2, TP53, TP63,TRAF7, TSC1, TSC2, TSHR, U2 AF1, VHL, XRCC2, XRCC3.

## Results

The clinicopathological and molecular profiles of the six cases are summarized in Table 2.

**Table 2:** Clinicopathological features and molecular profiles of study cases

	Patient 1	Patient 2	Patient 3	Patient 4	Patient 5	Patient 6
Age at diagnosis	68	57	66	46	64	60
Diagnosis at first presentation	Cervical punch biopsy	Cervical punch biopsy	Multiple biopsies	Cervical punch biopsy	Cervical punch biopsy	Cervical punch biopsy
Histology	Keratinizing Infiltrative-destructive pattern Amphophilic cytoplasm Extensive necrosis with comedo features Severe lymphocytic host response Precursor lesion was observed	Keratinizing Infiltrative-destructive pattern Amphophilic cytoplasm Extensive necrosis Severe lymphocytic host response Precursor lesion was not observed	Keratinizing Infiltrative-destructive pattern Amphophilic cytoplasm and pseudokoilocytic appearance Extensive necrosis with dirty necrosis Severe lymphocytic host response Precursor lesion was not observed	Non-keratinizing Infiltrative-destructive pattern Large clear and eosinophilic cytoplasm Extensive necrosis Moderate lymphocytic host response Precursor lesion was not observed	Keratinizing Infiltrative pattern Large clear and eosinophilic cytoplasm Necrosis was not observed Moderate lymphocytic host response Precursor lesion was observed	Keratinizing Downward tongue-like proliferation with focal invasion Large clear and eosinophilic cytoplasm Necrosis was not observed No lymphocytic host response Precursor lesion was not observed
Immunohistochemistry	p16^Ink4a^: Negative in both precursor lesion and invasive area p53: Mutant(overexpression) in bothprecursor lesion and invasive areas HER2 score: + 3	p16^Ink4a^: Negative p53: Wild Type HER2 score: 0	p16^Ink4a^: Negative p53: Wild Type HER2 score: 0	p16^Ink4a^: Negative p53: Wild Type HER2 score: 0	p16^Ink4a^: Negative in both precursor lesion and invasive area p53: Wild Type in both precursor lesion and invasive areas HER2 score: 0	p16^Ink4a^: Negative p53: Wild Type HER2 score: 0
Somatic Gene Mutation	TP53 mutation, ERBB2 copy number amplification	PIK3 CA mutation	PIK3 CA mutation	CTNNB1 mutation	None (Low key mutation:TP53 missense alteration 4%)	TERT promoter mutation
PD-L1	10% of tumor cells, 1% of tumor-associated immune cells (multifocal weak) CPS: 11	90% of tumor cells, 90% of tumor-associated immune cells (diffuse strong) CPS: 180	70% of tumor cells, 50% of tumor-associated immune cells (diffuse strong) CPS:120	2% of tumor cells, 1% of tumor-associated immune cells (weak) CPS: 2.5	5% of tumor cells, 1% of tumor-associated immune cells (weak) CPS: 10.5	Not detected CPS: 0
FIGO Stage	IIB	IIIC1 (clinical stage)	IVA (pelvic exenteration)	IVB (clinical stage)	IIB	N/A
Treatment	Radical hysterectomy Chemoradiotherapy	Chemoradiotherapy	Radical hysterectomy Chemoradiotherapy Pelvic exenteration	Chemoradiotherapy	Radical hysterectomy Chemoradiotherapy	Chemoradiotherapy
Follow-up	DOD 24 mo Recurrence not observed	Alive 15 mo w/o disease	DOD 50 mo Recurrence Site: Pelvis (Urinary Bladder24 mo after initial diagnosis)	DOD 2 mo Brain metastasis at the presentation	DOD 45 mo Recurrence Site: Vaginal cuff (17 mo after initial diagnosis)	DOD 6 mo Recurrence not observed

*DOD* died of disease, *w/o* without, *mo* month, *CPS* Combined positive score

### Patient 1 (TP53 mutation and ERBB2 amplification)

**Fig. 1:**
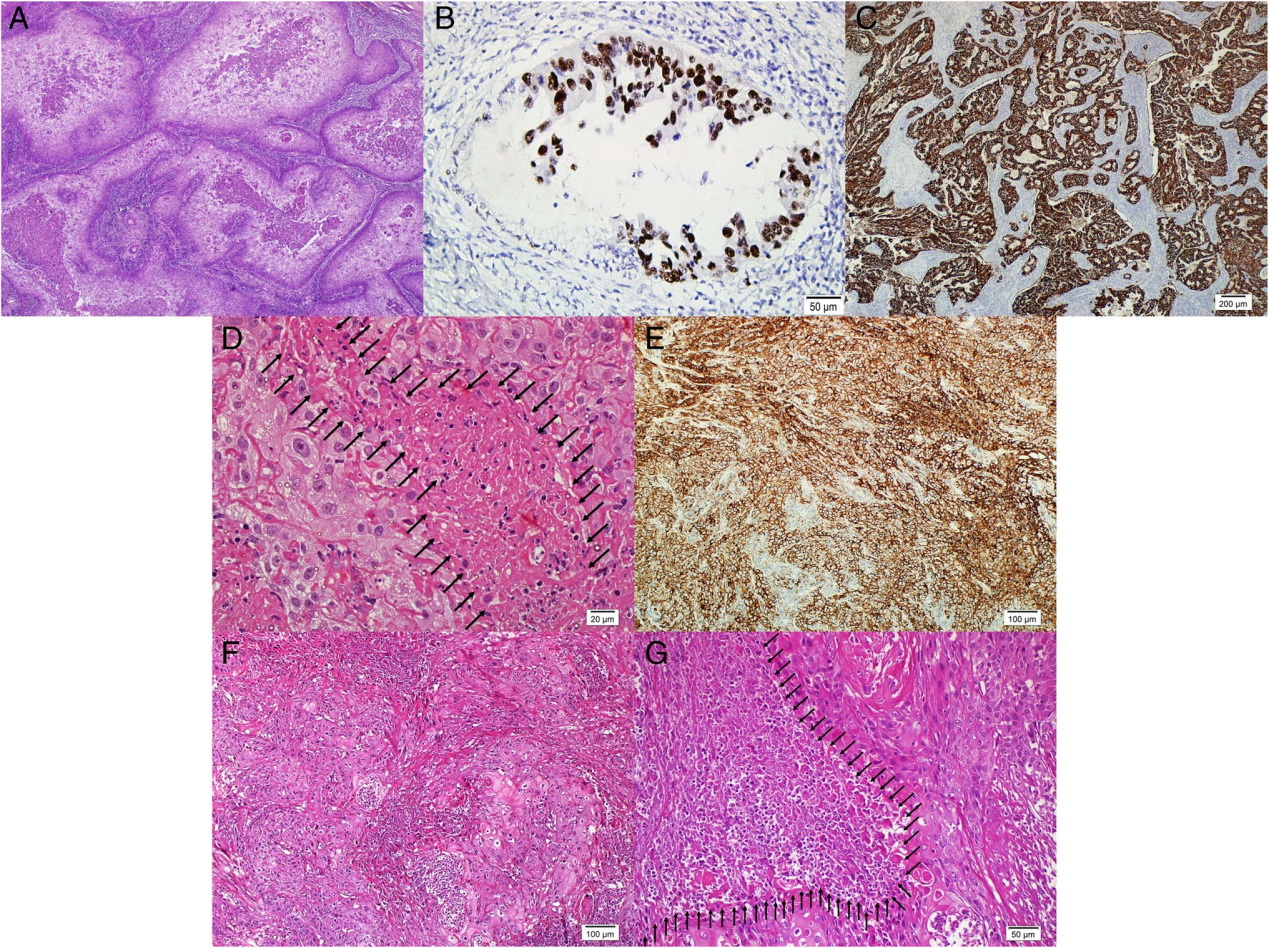
**A** Squamous cell carcinoma exhibiting extensive necrosis with comedo features (Patient 1, H&E, × 100). **B** Overexpression in the precursor lesion (right) showing transition with the benign endocervical epithelium(left) within endocervical gland (Patient 1, p53, × 200). **C** Score 3 positivity in the invasive area (Patient 1, HER2, × 40). **D** Squamous cell carcinoma with extensive necrosis (Patient 2, Arrows, H&E, × 400). **E** Diffuse, strong membranous positivity (Patient 2, PD-L1, × 100). **F** Squamous cell carcinoma with infiltrative-destructive pattern (Patient 3, H&E, × 100). **G** Squamous cell carcinoma exhibiting extensive necrosis with dirty features (Patient 3, Arrows, H&E, × 200)

A 68-year-old female underwent radical hysterectomy following a diagnosis of malignant tumor based on a cervical punch biopsy performed at another institution. A 3 cm tumor encompassing the entire cervix was identified. Histopathological examination revealed pronounced keratinization, an infiltrative-destructive growth pattern with amphophilic cytoplasm, and extensive necrosis with comedo features. A precursor lesion was not observed in the surface squamous epithelium, but a small precursor lesion arising from deeply located endocervical glands was identified. It was composed of atypical undifferantiated cells with 2–5 cell layer thickness showing sharp transition with the benign endocervical epithelium (Fig. 1A, B and Supplementary Fig. 1C). A severe lymphocytic host response was present (Table 3).

**Table 3:** Studies analyzing HPV-Independent squamous cell carcinoma of cervix

	Age	Histology	Immunohistochemistry	Somatic Gene Mutation	PD-L1	FIGO Stage	Outcome	HR HPV Analysis
Ikenberg et al. [1995]	N/A	N/A	p16: N/A p53: Abnormal 2/13 Wild type: 11/13 HER2: N/A	TP53 mutation (2/13)	N/A	N/A	N/A	Southern blot hybridisation and PCR
Nicolas et al. [2019]	38–85 Median Age: 59	Keratinizing (1/12) Non-keratinizing (10/12) Sarcomatoid (1/12) Precursor lesion: N/A	p16: 7/12 Block positive 5/12 Negative p53: Wild Type(3/12) Abnormal (not specified) (9/12) HER2 score: N/A	N/A	N/A	IB1-IVA	ANED (2/12) DOD (5/12) AWD (5/12)	INNO-Lipa Genotyping Extra II kit
Regauer et al. [2022]	36–65 Median Age: 51.5	Keratinizing (6/6) Precursor lesion: 6/6	p16: 1/6 Block positive both precursor lesion and invasive component 1/6 Block positive in precursor lesion, negative in the invasive component 4/6 Negative both in the precursor lesion and invasive component p53: Wild Type(5/6) Abnormal (overexpression) (1/6) HER2 score: N/A	Patient 1,2,5: None Patient 3: GNAS Patient 4: PIK3 CA, STK11 Patient 6: TP53	N/A	IA-IIIB	ANED: 7/9 Recurrence:1/9 DOD: 2/9	Aptima HPV Assay Cobas HPV test
Regauer et al. [2023]	63–74 (Median Age:65)	Keratinizing: 2/3 Non-keratinizing: 1/3 Precursor lesion: 3/3	p16: Negative both in the precursor lesion and invasive component 3/3 P53: Abnormal 1/3 (overexpression both in the precursor lesion and invasive component) 2/3: Wild Type (both in the precursor lesion and invasive component) HER2 score: N/A	Patient 1: TP53, SMARCB1/INI1 Patient 2: PIK3 CA, EGFR, SMARCB1/INI1 Patient 3: PIK3 CA, FGFR3, MET Low key mutation in: TP53, ABL,PTEN low key mutation	N/A	N/A	N/A	The CHIPRON HPV3.5LCD-array
Stolnicu et al. [2023]	37–88 (Median Age: 72,5)	Keratinizing: 6/10 Non-keratinizing: 3/10 Warty: 1/10 Precursor lesion: 5/10	p16: Negative (10/10) p53: Abnormal 2/10 (null type) Wild type 8/10 HER2 score: N/A	N/A	N/A	IA1-IVB	ANED:6/10 DOD: 3/10 AWD:1/10	ISH
Horn et al. [2025]	45–83 (Median Age: 73,5)	Keratinizing: 4/4 Non-keratinizing: 0/4 Precursor lesion: 3/4	p16: Negative both in the precursor lesion and invasive component (3/4) and negative in invasive component (1/4) p53: Abnormal 4/4 (overexpression both in the precursor lesion and invasive component 3/4, null type in invasive component 1/4) HER2: N/A	TP53 mutation (4/4)	N/A	IIA-IVA	ANED: 1/4 DOD: 2/4 Died non-tumor related: 1/4	The Vision Array HPV Chip
Stolnicu et al. [2025]	36–88 (Median Age: 68)	Keratinizing: 32/49 Non-keratinizing: 17/49 Precursor lesion: 20/49	p16: Negative both in the precursor lesion (20/20) and invasive component (49/49) p53:Abnormal in the invasive component 21/49(oveerexpression 16/21, null type 5/21) Wild type: 24/49 Unknown: 4/49 Abnormal in the precursor lesion 11/20 (8/11 overexpression, 3/11 Null type) Wild type or not tested in the precursor lesion: 9/20 HER2 score: N/A	N/A	N/A	I-IV	ANED: N/A Recurrence: 15/33 DOD: 23/49	ISH and PCR Based technique (INNO LIpa25, ANYPLEX II HPV28, SPf-10, APTIMA)
Kir et al	46–68 (Median Age:62)	Keratinizing: 5/6 Non-keratinizing: 1/6 Precursor lesion: 2/6 Extensive necrosis: 4/6 (Comedo features: 1/4 Dirty features: 2/4) Infiltrative-destructive growth pattern: 4/6 Infiltrative pattern: 1/6 Lymphocytic host response: Severe: 3/6 Moderate: 2/6 None: 1/6	p16: Negative in the precursor lesion (2/2) and invasive component 6/6 p53: Abnormal in the invasive component: 1/6(overexpression) Wild type in the invasive component: 5/6 Abnormal in the precursor lesion: 1/2 (overexpression) Wild type in the precursor lesion: 1/2 HER2 score: + 3 (1/6) 0 (5/6)	Patient 1: TP53 mutation, ERBB2 copy number amplification Patient 2: PIK3 CA Patient 3: PIK3 CA Patient 4: CTNNB1 mutation Patient 5: None (TP53 low key mutation) Patient 6: TERT promoter mutation	Patient 1: 10% of tumor cells, 1% of tumor-associated immune cells Patient2,3: Diffuse strong both in tumor cells and tumor associated immune cells Patient 4: 2% of tumor cells and 1% of tumor-associated immune cells Patient 5: 5% of tumor cells and 1% of tumor-associated immune cells Patient 6: None	IIB-IVA	ANED: 1/6 Recurrence: 2/6 DOD: 5/6	Aptima HPV Assay

*N/A* Not available, *ANED* Alive no evidence of disease, *DOD* Died of disease, *AWD *Alive with disease, *ISH* in-situ hybridization

Immunohistochemically,both the precursor lesion and the invasive tumor exhibited patchy p16 staining, and overexpression with p53. PD-L1 expression was observed in 10% of tumor cells and 1% of tumor-associated immune cells. CPS was 11 (Supplementary Fig. 1A,B,D)**.** HER2 was scored as 3 + (Fig. 1C). The high-risk HPV Aptima assay was negative.

Next-Generation Sequencing (NGS) identified a c.652_654 dup p.Val218 dup inframe duplication in the TP53 gene (allele fraction: 12%). ERBB2 gene amplification (copy number: 4.14) was also detected. The patient, staged as FIGO IIB, underwent chemoradiotherapy but passed away 24 months after diagnosis.

### Patient 2 (PIK3 CA mutation)

A 57-year-old female underwent cervical biopsy for a mass detected during gynecological examination. MRI revealed a 5 cm tumor and metastatic pelvic lymph nodes. Histopathological evaluation showed a keratinizing tumor with an infiltrative-destructive growth pattern, amphophilic cytoplasm, and extensive necrosis**,** without evidence of precursor lesions (Fig. 1D). A severe lymphocytic host response was observed.

Immunohistochemically, CK 5/6 was diffusely positive, p16 was negative, and p53 expression was wild-type (Supplementary Fig. 2A-D). PD-L1 positivity was noted in > 90% of tumor cells and tumor-associated immune cells. CPS was 180 (Fig. 1E). HER2 expression was scored as 0. The high-risk HPV Aptima assay was negative.

NGS revealed a c.1357G > A p.Glu453Lys missense mutation in the PIK3 CA gene (allele fraction: 11%). The patient, staged as FIGO IIIc1, underwent chemoradiotherapy and remained recurrence-free after 15 months of follow-up.

### Patient 3 (PIK3 CA mutation)

A 66-year-old female previously underwent radical hysterectomy for HPV-independent SCC at another center. 24 months after diagnosis due to pelvic (urinary bladder) recurrence, pelvic exenteration was performed at our institution. Histopathological evaluation revealed a keratinizing tumor with an infiltrative-destructive pattern, amphophilic cytoplasm, pseudokoilocytic appearance, and extensive necrosis with dirty features (Fig. 1F,G). Precursor lesion was not identified. A severe lymphocytic host response was noted.

Immunohistochemically, CK 5/6 was diffusely positive, p16 was negative, and p53 expression was wild-type. PD-L1 positivity was observed in 70% of tumor cells and 50% of tumor-associated immune cells. CPS was 120 (Supplementary Fig. 3A-D). HER2 expression was scored as 0. The high-risk HPV Aptima assay was negative.

NGS detected a c.163G > A p.Glu545Lys missense mutation in the PIK3 CA gene (allele fraction: 17%). The patient, staged as FIGO IVa, underwent chemoradiotherapy but passed away 50 months after the initial diagnosis.

### Patient 4 (CTNNB1 mutation)

**Fig. 2:**
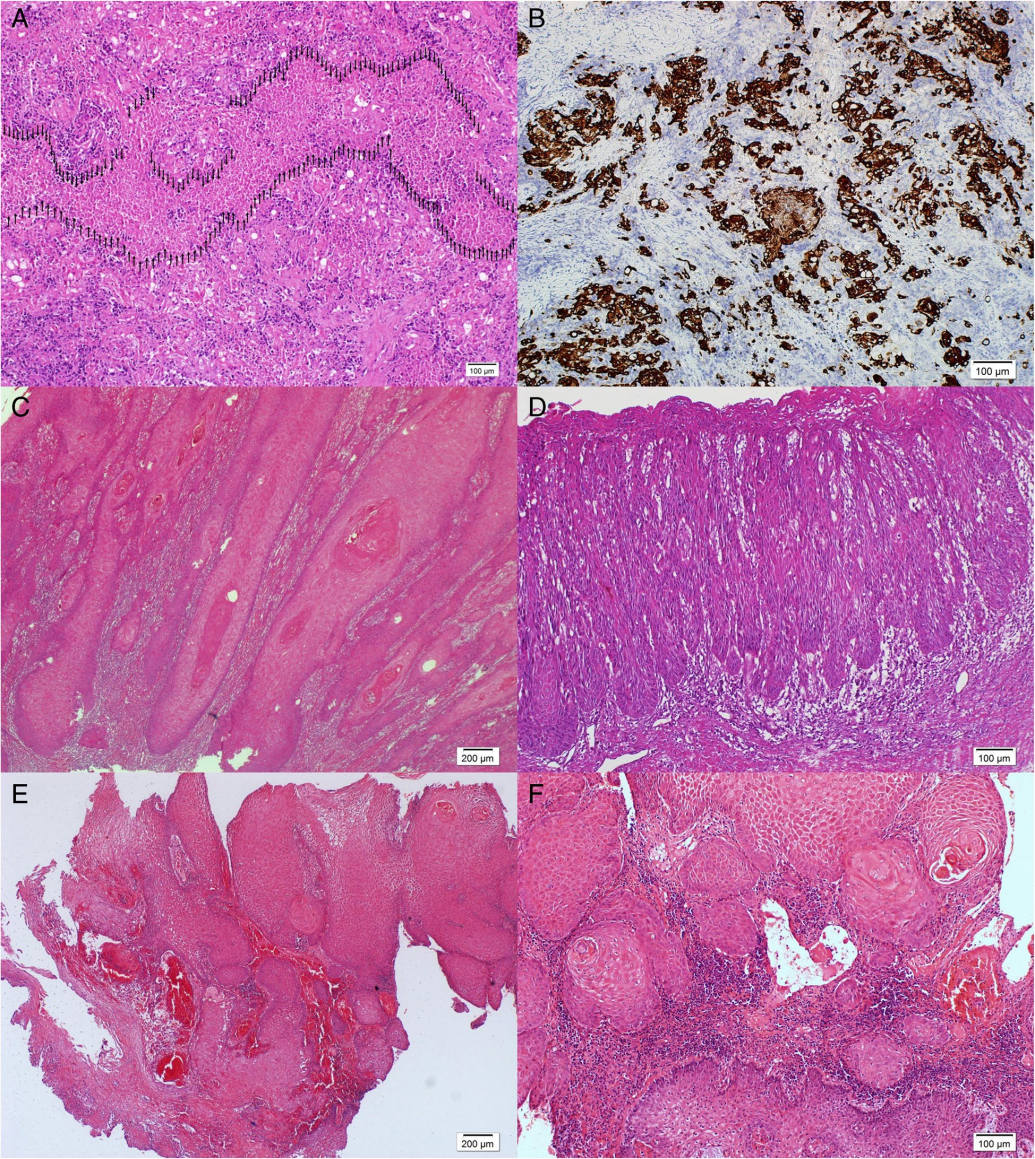
**A** Squamous cell carcinoma with infiltrative-destructive pattern, and extensive necrosis (Patient 4, Arrows, H&E, × 100). **B** Squamous cell carcinoma with infiltrative-destructive pattern (Patient 4, CK5/6, × 100). **C** Keratinizing squamous cell carcinoma with infiltrative growth pattern (Patient 5, H&E, × 40). **D** Precursor lesion showing d-VIN like morphology (Patient 5, H&E, × 100). **E** Well-differentiated keratinizing squamous cell carcinoma (Patient 6, H&E, × 40). **F** Squamous cell carcinoma with downward proliferation and invasion (Patient 6, H&E, × 100)

A 46-year-old female presented with vaginal bleeding and underwent cervical punch biopsy. Histopathological analysis revealed a non-keratinizing tumor with an infiltrative-destructive growth pattern, large cells with eosinophilic cytoplasm, and extensive necrosis (Fig. 2A and Supplementary Fig. 4 A,B). No precursor lesion was observed. A moderate lymphocytic host response was noted.

Immunohistochemically, CK5/6 was diffusely positive, patchy p16 staining was observed, and p53 expression displayed a wild-type pattern (Supplementary Fig. 4 C,D). PD-L1 was positive in 2% of tumor cells and 1% of tumor-associated immune cells. CPS was 2.5. HER2 was scored as 0. The high-risk HPV Aptima assay was negative.

NGS identified a c.110 C > T p.(Ser37Phe) missense mutation in exon 3 of the CTNNB1 gene (allele fraction: 26.7%). The patient, staged as FIGO IVB with brain metastases at the presentation, underwent chemoradiotherapy but passed away 2 months after diagnosis.

### Patient 5

A 64-year-old female previously diagnosed with HPV-independent SCC underwent radical hysterectomy at our institution. A 6 cm tumor covering the cervix was identified. Histopathological examination revealed a keratinizing morphology with eosinophilic cytoplasm and an infiltrative growth pattern (Fig. 2C). Necrosis was not observed. Precursor lesion was observed at the surface squamous epithelium of ectocervix. Precursor area of surface epithelium was highly differantiated and keratinizing similar to differantiated vulvar intraepithelial neoplasia, including hyperkeratosis, parakeratosis, elongation of rete ridges, significant nuclear atypia and mitotic activity at the basal and parabasal cells. Premature squamatization with large cells with pink cytoplasm in suprabasal cell layers was prominent (Fig. 2D). A mild lymphocytic host response was observed.

Immunohistochemically, p16 was negative in both precursor lesion and invasive area, and p53 expression was wild-type in both precursor lesion and invasive area (Supplementary Fig. 5 A-D). PD-L1 positivity was noted in 5% of tumor cells and 1% of tumor-associated immune cells. CPS was 10.5**.** HER2 expression was scored as 0. The high-risk HPV Aptima assay was negative.

NGS identified a c.347 C > T p.Ser116Phe missense mutation in the TP53 gene (allele fraction: 4%). The patient, initially staged as FIGO IIB, underwent chemoradiotherapy. A vaginal cuff recurrence was observed 17 months after the initial diagnosis; however, the patient passed away 45 months post-diagnosis.

### Patient 6 (TERT promoter mutation)

A 60-year-old female presented with a 3.8 cm cervical mass. Histopathological evaluation revealed a well-differentiated, keratinizing tumor with downward tongue-like proliferation and focal invasion (Fig. 2E, F). Necrosis, precursor lesion and lymphocytic host responses were not observed.

Immunohistochemically p16 was negative, and p53 expression was wild-type (Supplementary Fig. 6 A,B). PD-L1 was not expressed in tumor or tumor-associated immune cells. CPS was 0. HER2 expression was scored as 0. The high-risk HPV Aptima assay was negative.

NGS revealed a c.−124C > T (C228 T) variant in the TERT promoter region (allele fraction: 21%). The patient underwent chemoradiotherapy but passed away 6 months after diagnosis.

## Discussion

Despite growing awareness of HPV-independent SCC, research on its clinicopathological characteristics, molecular profiles, and potential therapeutic targets remains limited. This study provides some insights into HPV-independent cervical squamous cell carcinoma (SCC), a rare and aggressive form of cervical cancer. By comparing our findings with preliminary studies we examined the clinicopathological and molecular characteristics that distinguish HPV-independent SCC [3, 4, 14–18].

In our cohort of six cases, the median age at diagnosis was 62 years (range: 46–68). This finding aligns with the literature, where the median age for HPV-independent SCC ranges from 51.5 to 73.5 years [3, 4, 15–18]. These results support the observation that HPV-independent SCC predominantly affects older patients compared to HPV-associated SCC, suggesting a distinct carcinogenic pathway that likely develops later in life [1, 2].

In our study, five cases exhibited keratinizing morphology, while one was non-keratinizing. Extensive necrosis with or without comedo and dirty features was observed in 4 of 6 cases. Necrosis, frequently associated with the aggressive nature of HPV-independent SCC, was similarly reported by Stolnicu et al. [3]**.** In previous studies, Nicolás et al. reported predominantly non-keratinizing tumors, whereas all other studies, including current study, predominantly identified keratinizing morphology [3, 4, 15–18]. In our cohort, precursor lesions were observed in two cases, which is consistent with findings reported in the literature [3, 4, 16–18]. Precursor lesions were observed in 6/6, 3/3, 5/10, 3/4, and 17/49 of the cases in studies reporting the presence of precursor lesions. Patient 1 exhibited a precursor lesion originating within deeply located endocervical glands. Immunohistochemically, it was negative for p16 and showed overexpression of p53. In Patient 5, the precursor area of the surface epithelium was highly differentiated and keratinizing, resembling differentiated vulvar intraepithelial neoplasia. Immunohistochemically, the precursor lesion was negative for p16 and showed a wild-type p53 expression pattern.

All six cases were p16-negative in both precursor lesions and invasive components, a hallmark of HPV-independent SCC, consistent with the majority of studies in the literature reporting similar findings [3, 16–18]. Regarding p53 expression, one case in our cohort exhibited overexpression (abnormal) in both the precursor lesion and invasive components, while the remaining five cases demonstrated a wild-type expression pattern in both precursor lesion and invasive areas, consistent with the findings of Regauer et al. and Stolnicu et al. [3, 4, 16, 18].

TP53 and PIK3 CA mutations were identified in our cohort, consistent with findings from previous studies[4, 14]. Furthermore, mutations in STK11, GNAS, EGFR, and SMARCB1 were observed [14, 16]. These overlapping genetic alterations suggest that TP53 and PIK3 CA mutations may play a critical role in HPV-independent cervical SCC carcinogenesis, which have also been reported in HPV-independent vulvar SCC cases in the literature [19]. Additionally our study identified CTNNB1 and TERT promoter mutations, which were not observed in any of the other studies reviewed. These findings highlight potential novel genetic drivers in HPV-independent SCC. Our finding of CTNNB1 and TERT promoter mutations expands the known mutational landscape of HPV independent cervical tumors. Moreover, TERT promoter mutations have been observed in vulvar SCCs and HPV-independent penile SCCs [20, 21]. In our case with a TP53 mutation, we also observed a high ERBB2 copy number and a HER2 immunohistochemistry score of 3 +. Recent studies on the molecular classification of endometrium cancer have highlighted the association between p53 mutations and HER2 amplification [22].

Recent reports analyzing the PD-L1 expression of cervical squamous cell carcinomas did not take into consideration of HPV status [23, 24]. Food and drug administration has recently approved PD-L1 blockers in the treatment of advanced PD-L1 positive cervical cancer with a CPS cut-off ≥ 1 [24]. In categorical analysis 36% had less than 1% PD-L1 and negative result. 64% expressed PD-L1 antibody (≥ 1% PD-L1 expression). There was diffuse PD-L1 expression (50% or more) in 23% of the cases [24]. Monsrud et al. immunostained for PD-L1 in 73 cases of cervical squamous cell carcinoma with CPS ≥ 1 and ≥ 10 as the cut-off, PD-L1 was positive in 71.2% and 31.5% of cases respectively. PD-L1 expression varied within our cohort, with a minimum focal expression and with at least 2.5 CPS score observed in five out of six cases in both tumor cells and tumor-associated immune cells. This finding suggests that PD-L1 expression could serve as a promising biomarker for immunotherapy in HPV- independent SCC. Further research is needed to evaluate the efficacy of immune check point inhibitors in this subset of cervical cancer.

The majority of patients in our cohort presented with advanced FIGO stages (IIB–IVB), consistent with previous reports [3, 4, 15–18]. This finding highlights the aggressive nature of HPV-independent SCC and the challenges associated with early detection. That underscores the aggressive nature of HPV-independent SCC and the challenges in early detection. In our cohort, five out of six patients succumbed to the disease, with recurrence occurring in two of them, while one remained alive with no evidence of disease. These outcomes reflect the poor prognosis associated with HPV-independent SCC, as noted in the literature. Nicolás et al. reported significantly worse disease-free and overall survival in HPV-independent SCC compared to HPV-positive cases [15]. Similarly, Stolnicu et al. observed a 51.2% mortality rate and a 58.1% recurrence rate [18]. These consistently poor outcomes emphasize the urgent need for novel therapeutic strategies for this aggressive malignancy.

## Conclusion

While the small number of cases is a limitation of our study, our findings align with the literature in demonstrating that HPV-independent cervical SCC predominantly affects older patients. Histologically, keratinization, an infiltrative-destructive growth pattern, and extensive necrosis were common features observed in most cases. Advanced-stage presentation was typical, and the majority of patients experienced poor outcomes, often succumbing to the disease within a short follow-up period.

Genetically, our study corroborates previous reports of TP53 and PIK3 CA mutations in HPV-independent cervical SCC. Additionally, we identified novel genetic alterations, including CTNNB1 and TERT promoter mutations, as well as HER2 amplification, which had not been previously reported in this subtype. HER2 amplification, in particular, may represent a potential target for molecularly directed therapies. Furthermore, the detection of PD-L1 expression with at least CPS score of 2,5 in five of six patients may suggest the potential utility of immunotherapy in treating HPV-independent cervical SCC**.** Given the absence of specific treatment guidelines for this rare and aggressive malignancy, future research should prioritize identifying effective therapeutic approaches, including targeted molecular treatments and immunotherapy, to improve patient outcomes.

## Supplementary Information

Below is the link to the electronic supplementary material.

Supplementary Material 1. Figure 1 (Patient 1) A: Patchy staining in the invasive area (p16, × 200). B: Overexpression in the invasive area (p53, × 200). C: Precursor lesion originating in a deeply located endocervical gland (Arrows, H&E, × 100). D: Patchy staining in the precursor lesion (p16, × 200). (JPG 2.77 MB)

Supplementary Material 2. Figure 2 (Patient 2) A: Squamous cell carcinoma (H&E, × 100). B: Squamous cell carcinoma with infiltrative-destructive pattern (CK5/6, × 100). C: Negative staining (p16 × 100). D: Wild type staining (p53, × 200). (JPG 2.97 MB)

Supplementary Material 3. Figure 3 (Patient 3) A: Squamous cell carcinoma with infiltrative-destructive pattern (CK5/6, × 100). B: Negative staining (p16, × 100). C: Wild type staining (p53, × 100). D: Diffuse membranous staining (PD-L1, × 100). (JPG 2.67 MB)

Supplementary Material 4. Figure 4 (Patient 4) A: Squamous cell carcinoma (H&E, × 40). B: Squamous cell carcinoma with extensive necrosis (H&E, × 200). C: Patchy staining (p16, × 200). D: Wild type staining in invasive area (p53, × 200). (JPG 2.32 MB)

Supplementary Material 5. Figure 5 (Patient 5) A: Patchy staining in invasive area (p16, × 200). B: Wild type staining in invasive area (p53, × 200). C: Patchy staining in the precursor lesion (p16, × 200). D: Wild type staining in the precursor lesion (p53, × 200). Figure 6 (Patient 6) A: Negative staining (p16, × 100). B: Wild type staining (p53, × 100). (JPG 1.38 MB)
