# Supplementary figures and images for: HPV-independent squamous sell carcinoma of cervix: a clinicopathological, immunohistochemical, and molecular analysis of six cases
Source: Virchows Arch. 2025 May 10;487(4):775–86. doi: 10.1007/s00428-025-04113-6 (PMC12546508; doi:10.1007/s00428-025-04113-6)

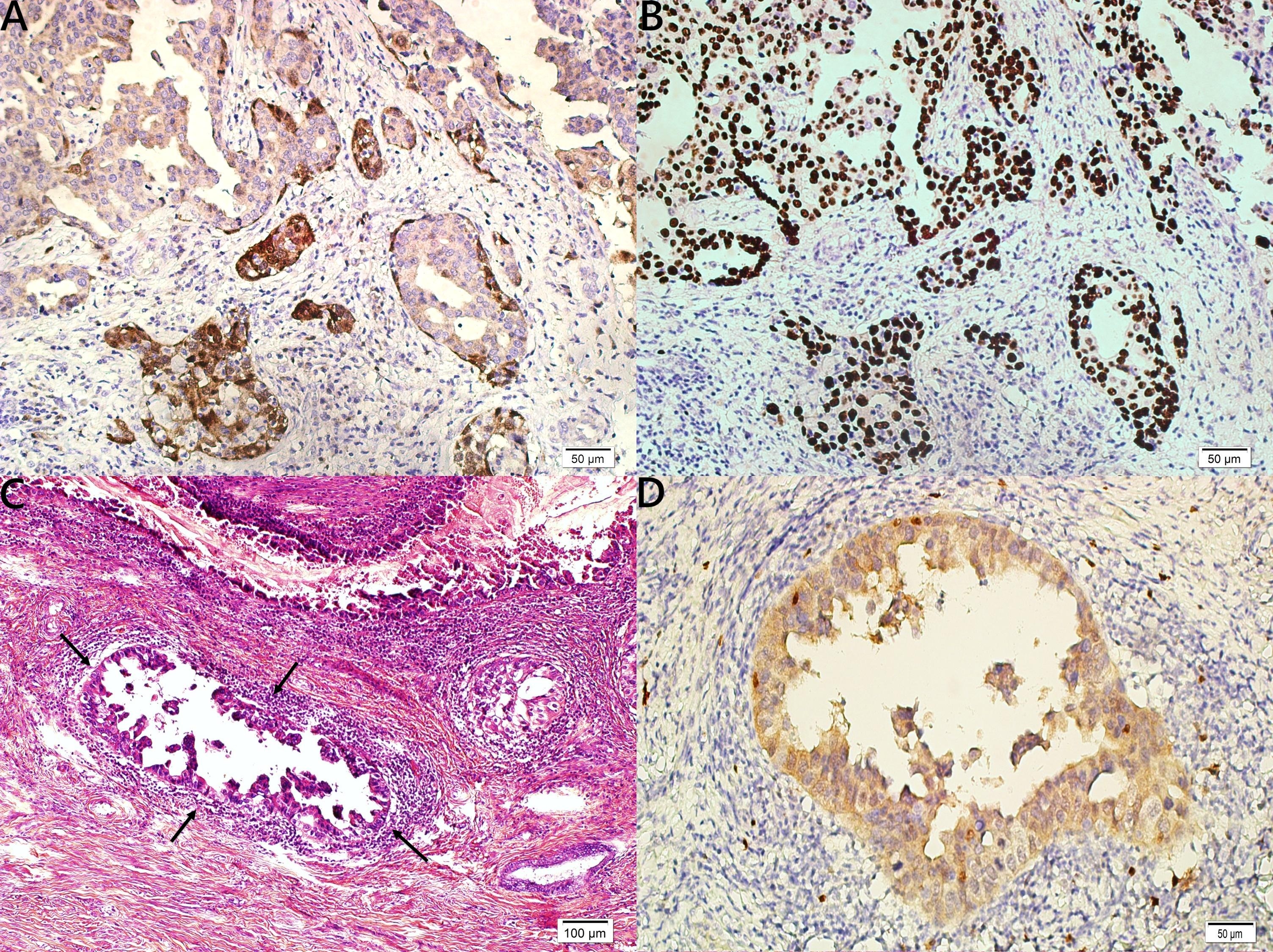

Supplement: Supplementary file 1 — Supplementary Material 1. Figure 1 (Patient 1) A: Patchy staining in the invasive area (p16, × 200). B: Overexpression in the invasive area (p53, × 200). C: Precursor lesion originating in a deeply located endocervical gland (Arrows, H&E, × 100). D: Patchy staining in the precursor lesion (p16, × 200). (JPG 2.77 MB) [file 428_2025_4113_MOESM1_ESM.jpg]

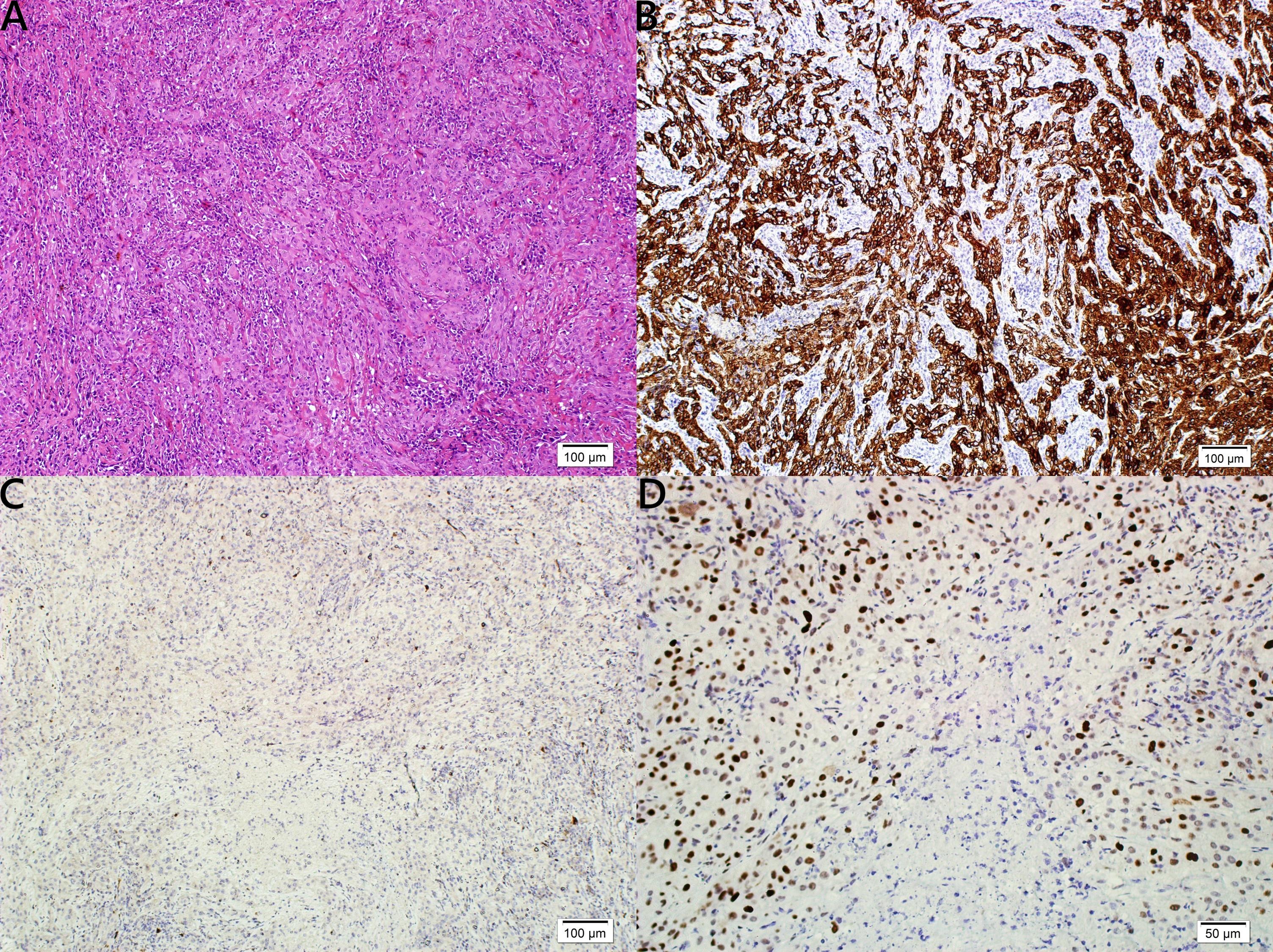

Supplement: Supplementary file 2 — Supplementary Material 2. Figure 2 (Patient 2) A: Squamous cell carcinoma (H&E, × 100). B: Squamous cell carcinoma with infiltrative-destructive pattern (CK5/6, × 100). C: Negative staining (p16 × 100). D: Wild type staining (p53, × 200). (JPG 2.97 MB) [file 428_2025_4113_MOESM2_ESM.jpg]

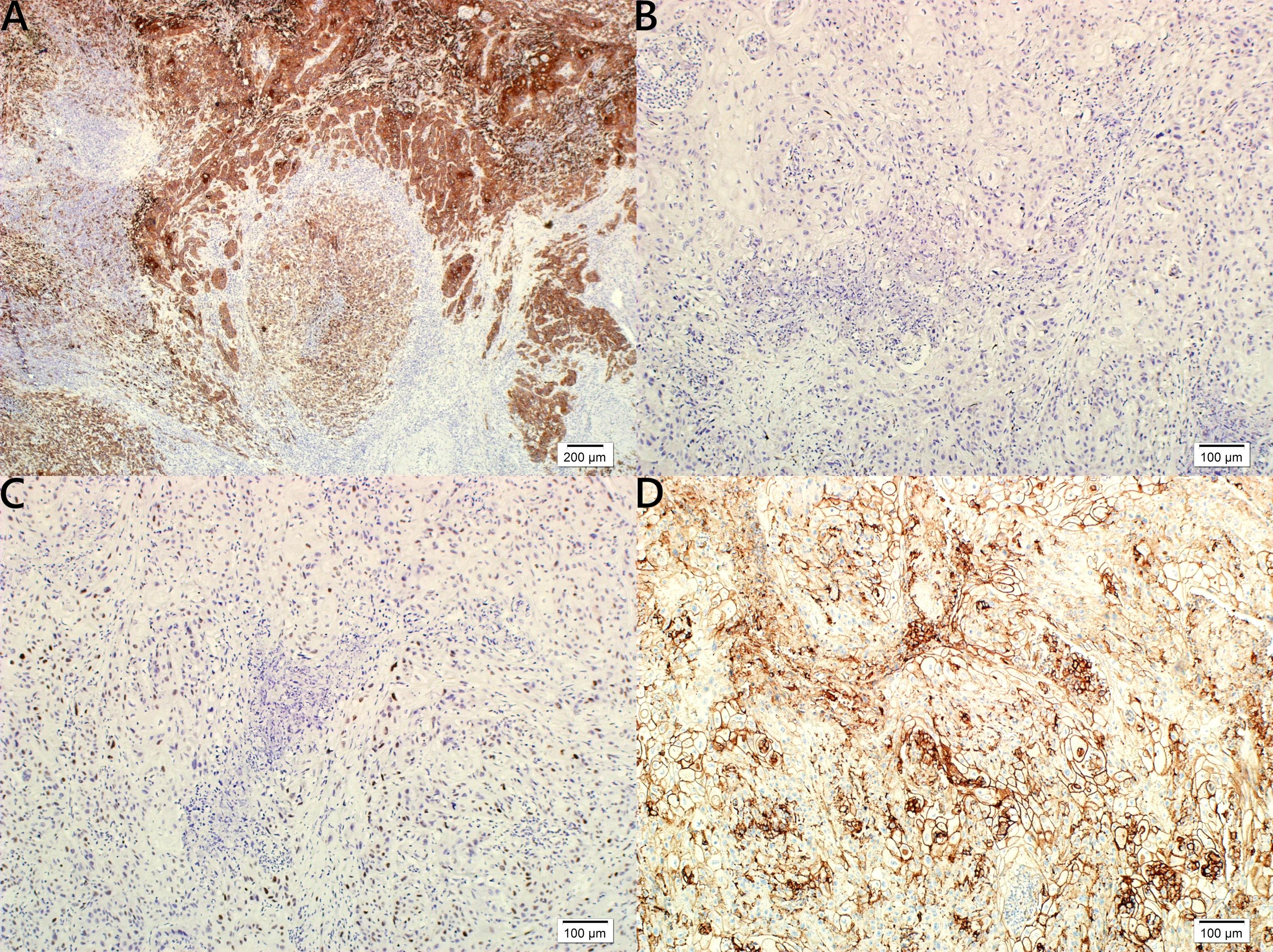

Supplement: Supplementary file 3 — Supplementary Material 3. Figure 3 (Patient 3) A: Squamous cell carcinoma with infiltrative-destructive pattern (CK5/6, × 100). B: Negative staining (p16, × 100). C: Wild type staining (p53, × 100). D: Diffuse membranous staining (PD-L1, × 100). (JPG 2.67 MB) [file 428_2025_4113_MOESM3_ESM.jpg]

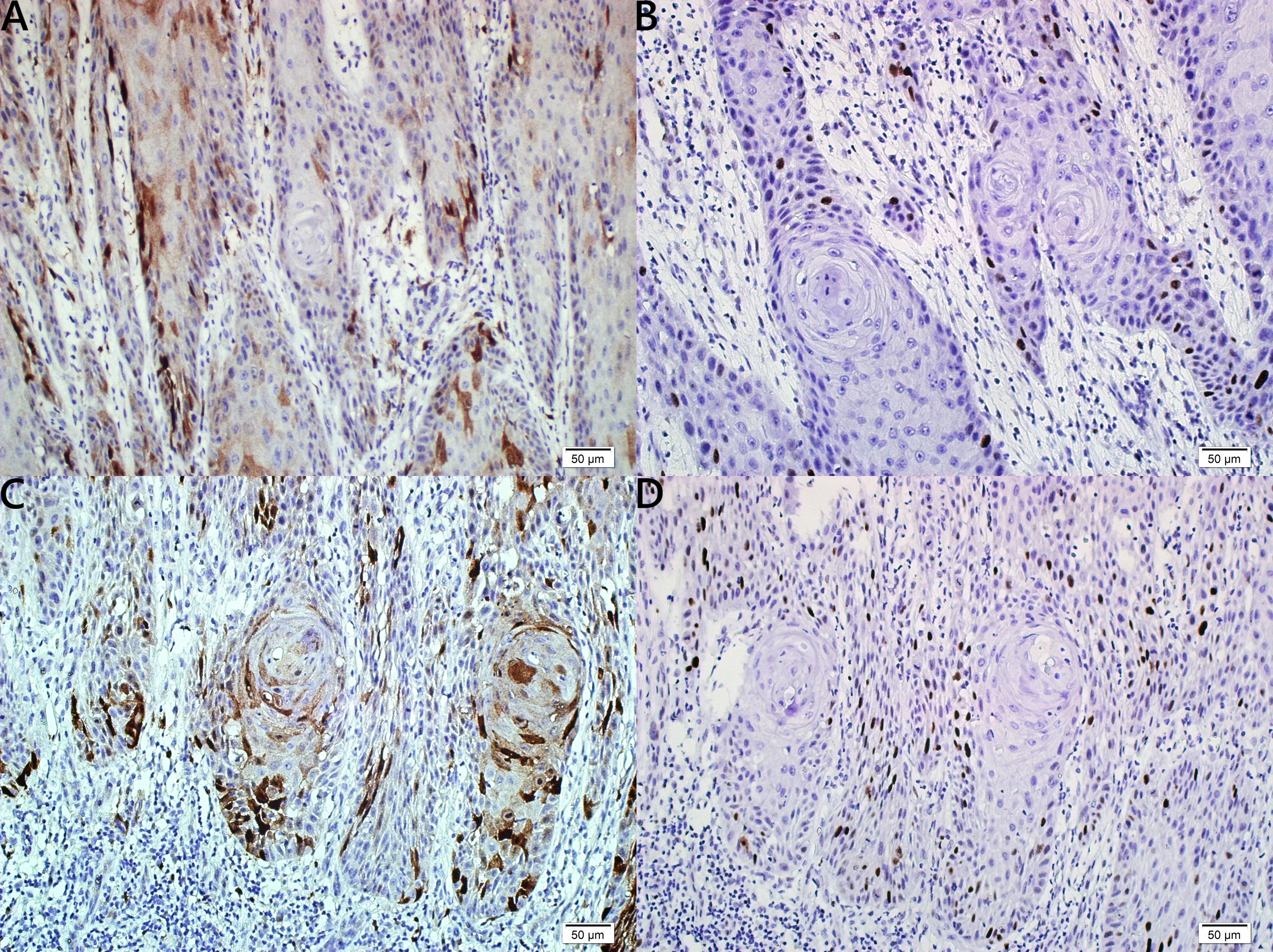

Supplement: Supplementary file 4 — Supplementary Material 4. Figure 4 (Patient 4) A: Squamous cell carcinoma (H&E, × 40). B: Squamous cell carcinoma with extensive necrosis (H&E, × 200). C: Patchy staining (p16, × 200). D: Wild type staining in invasive area (p53, × 200). (JPG 2.32 MB) [file 428_2025_4113_MOESM4_ESM.jpg]

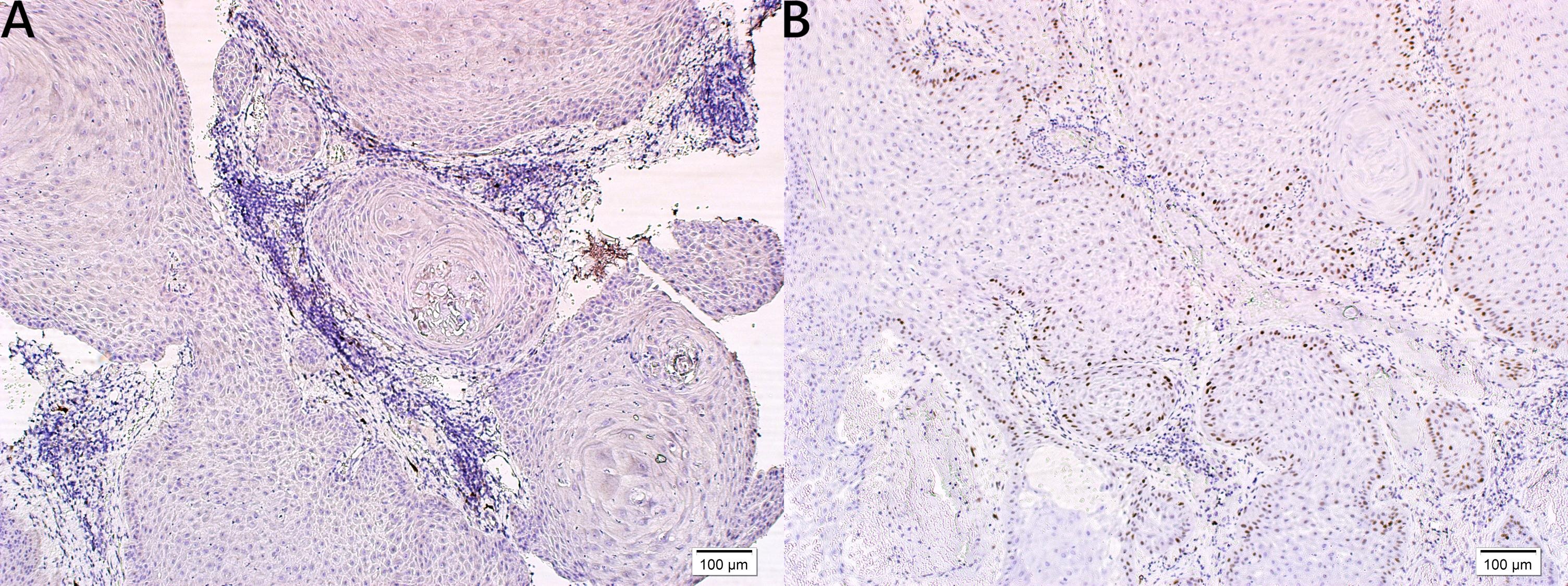

Supplement: Supplementary file 5 — Supplementary Material 5. Figure 5 (Patient 5) A: Patchy staining in invasive area (p16, × 200). B: Wild type staining in invasive area (p53, × 200). C: Patchy staining in the precursor lesion (p16, × 200). D: Wild type staining in the precursor lesion (p53, × 200). Figure 6 (Patient 6) A: Negative staining (p16, × 100). B: Wild type staining (p53, × 100). (JPG 1.38 MB) [file 428_2025_4113_MOESM5_ESM.jpg]
